# Response of Growth Performance, Blood Biochemistry Indices, and Rumen Bacterial Diversity in Lambs to Diets Containing Supplemental Probiotics and Chinese Medicine Polysaccharides

**DOI:** 10.3389/fvets.2021.681389

**Published:** 2021-06-24

**Authors:** Huan Chen, Beibei Guo, Mingrui Yang, Junrong Luo, Yiqing Hu, Mingren Qu, Xiaozhen Song

**Affiliations:** ^1^Jiangxi Province Key Laboratory of Animal Nutrition/Engineering Research Center of Feed Development, Jiangxi Agricultural University, Nanchang, China; ^2^College of Animal Science, South China Agricultural University, Guangzhou, China; ^3^Jiangxi Provincial Key Laboratory for Animal Health, Institute of Animal Population Health, College of Animal Science and Technology, Jiangxi Agricultural University, Nanchang, China

**Keywords:** Chinese medicine polysaccharide, probiotics, growth performance, rumen fermentation, rumen bacteria composition, lamb

## Abstract

This study aims to investigate the effects of probiotics and Chinese medicine polysaccharides (CMPs) on growth performance, blood indices, rumen fermentation, and bacteria composition in lambs. Forty female lambs were randomly divided into four groups as follows: control, probiotics, CMP, and compound (probiotics + CMP) groups. The results showed that probiotics treatment increased the concentrations of blood glucose (GLU) and immunoglobulin G (IgG) and enhanced rumen microbial protein contents but declined the value of pH in rumen fluid compared with the control (*P* < 0.05). Furthermore, supplementation with CMP enhanced the average daily gain (ADG) and the contents of IgA, IgG, and IgM in the serum but decreased the F:G ratio compared with the control (*P* < 0.05). Besides, both CMP and compound (probiotics + CMP) treatments decreased the ratio of acetic acid and propionic acid compared with the control (*P* < 0.05). High-throughput sequencing data showed that at the genus level, the relative abundance of *Veillonellaceae_UCG-001* in the probiotics group was increased, the relative abundance of *Succiniclasticum* and *norank_f__Muribaculaceae* in the CMP group were enhanced, and the relative abundance of *Ruminococcaceae_UCG-002* in the compound group was raised compared with the control (*P* < 0.05). In summary, supplementation with probiotics can promote rumen protein fermentation but decrease the diversity of bacteria in rumen fluid; however, CMP treatment increased the relative abundance of *Fibrobacteria*, changed rumen microbial fermentation mode, increased the immune function, and ultimately improved the growth performance.

## Introduction

Antibiotics have long been used for therapeutic and subtherapeutic purposes, including disease treatment, disease prevention, and growth performance (1). However, the increased use of antibiotics as a feed additive resulted in the emergence of drug residues in animal products and harm human health, leading to a ban on the use in many countries, such as China, EU, USA. Recently, research has begun to investigate more carefully the use of probiotics and herbal extracts and plant bioactives as an alternative approach to enhancing animal health and production efficiency (2).

Probiotics are defined as direct-fed microbials, including *yeast* (*Saccharomyces cerevisiae*), *Bacillus*, and *Lactobacillus*, which may lead to the creation of a microbial balance in the gastrointestinal tract and confer a health benefit for the host when administered in appropriate and regular quantities (3). A previous study revealed that probiotics could enhance the digestion of fiber and starch, promote volatile fatty acid (VFA) synthesis, and increase the average daily gain and feed conversion rate of cattle (4). Another report showed that adding *Lactobacillus acidophilus* to lambs' diet could increase the dry matter feed intake and daily weight gain and improve the digestibility of dry matter, crude protein, and crude fiber (5).

Chinese medicine polysaccharides are secondary metabolites produced by Chinese herbs metabolism, and it contains more than 10 degrees of polymerization (6, 7). A series of studies have shown that polysaccharides, including *Astragalus* polysaccharide and *Lycium barbarum* polysaccharide, can enhance immunity, are antioxidative, and can improve animal production performance (8, 9). *Astragalus* polysaccharide is the extract of *Astragalus membranaceus*, which is often used to enhance immunity in traditional Chinese medicine. The polysaccharide of *L. barbarum* is its major bioactive component and has been widely used as one of the main chemical components in a traditional Chinese medicine named Ningxia wolfberry. *Astragalus* polysaccharide has been reported to be a potential additive used in the vaccination of humans and animals so as to improve immune functions both in cellular and humoral immune responses (10). *Astragalus* polysaccharide was reported to enhance the antioxidant capacity while adding to the diet of goats (11). Furthermore, a recent report showed that *L. barbarum* polysaccharide (LBP) improved growth promotion and immunomodulation and could be used as an alternative for nutritive additive in broilers (12).

At present, probiotics and Chinese medicine polysaccharides have been used for immunity, growth performance, and metabolism in non-ruminants and ruminants. However, little information is available regarding their effects on rumen bacterial diversity and fermentation parameters of lambs. Rumen microbial ecosystems include a variety of strict anaerobes, protozoa, and archaea, which are in charge of the degradation and fermentation of most nutrients such as dietary fiber (13). Therefore, the purpose of this study is to investigate the effects of probiotics and Chinese medicine polysaccharides on growth performance, metabolism, rumen bacterial diversity, and fermentation parameters in lambs. These results will give helpful information on the effects and application of probiotics and Chinese medicine polysaccharides in ruminants farming.

## Materials and Methods

### Probiotics and Chinese Medicine Polysaccharides

Probiotics were donated by Nanchang University, which consists of *Bacillus licheniformis, Bacillus subtilis*, and *Lactobacillus plantarum* at a ratio of 1:1:0.5. The Chinese medicine polysaccharide (CMP) comes from the mixture of *L. barbarum* and *A. membranaceus* in the ratio of 2:1 and in which the content of polysaccharides was 114.7 mg/g.

### Animals and Experimental Design

This experiment was approved by the Committee for the Care and Use of Experimental Animals at Jiangxi Agricultural University (JXAULL-20190015). A total of 40 healthy female lambs (Chuanzhong black) with an average body weight of 21.69 ± 0.46 kg and the ages ranging 4–5 months were housed indoors in an individual pen (0.5 × 0.6 m^2^) with a leaky floor, which can keep the pen clean and reduce the labor cost of cleanliness. The feeding trial lasted for 60 days after a 10-day adaptation period. During the trial, a single-factor completely randomized design method was used, and all lambs were randomly divided into four treatments, namely, control (basal diet), probiotics (0.1% probiotics + basal diet), CMP (0.1% Chinese medicine polysaccharides + basal diet), and compound (0.1% probiotics + 0.1% Chinese medicine polysaccharides + basal diet), and each treatment contained 10 lambs. These lambs fed *ad libitum* with standard ration consisted mainly of peanut vine, corn, soybean meal, wheat bran, and mineral, which were formulated to meet the feeding standard of meat-producing sheep and goat of NY/T 816-2004 (Ministry of Agriculture of the People's Republic of China 2004), and the ingredient composition and nutrient levels are shown in Table 1. No antibiotic was included in the diet.

**Table 1:** Basic diet composition and nutritional status (air-dried basis)^a^, ^b^, ^c^.

**Ingredients**	**Content (%)**	**Nutrition levels^b^**	**Content (%)**
Peanut vine	50.00	DM	84.01
Corn	30.00	ME/(MJ/kg)	7.07
Soybean meal	11.00	CP	12.53
Wheat bran	4.00	Ca	1.20
CaHPO_4_	2.00	P	0.64
NaHCO_3_	1.50	NDF	33.03
NaCl	0.50	ADF	22.24
Premix	1.00	DE/(MJ/kg)	11.08
Total	100.00		

a *Premix contained 12,000 IU/kg of vitamin A, 5,000 IU/kg of vitamin D, 50 mg/kg of vitamin E, 40 mg/kg of Fe, 16 mg/kg of Cu, 70 mg/kg of Zn, 80 mg/kg of Mn, 0.3 mg/kg of Co, 0.8 mg/kg of I, and 0.3 mg/kg of Se*.

b *DM, dry matter; ME, metabolizable energy; CP, crude protein; NDF, neutral detergent fiber; ADF, acid detergent fiber; DE, digestible energy*.

c *Nutrition levels are values of measurement except that ME is value from a calculation*.

### Determination of the Growth Performance

Body weight of animals on an empty stomach was measured at 09:00 h at the beginning and end of the trial, and the daily feed intakes were recorded during the experimental period. Based on these data, average daily feed intake (ADFI), average daily gain (ADG), and the ratio of feed and gain (F:G) ratio were calculated.

### Serum Biochemistry Indices Analysis

On the 60th day, blood samples (10 ml each) were collected at 14:00 h from the jugular vein, using a vacuum blood collection tube (Nanjing Jiancheng Bioengineering Institute, Nanjing, China). Serum was obtained by immediate centrifugation (10 min, 4°C, 1,500 *g*) and kept at −80°C until analysis. The concentrations of serum total protein (TP), albumin (ALB), blood urea nitrogen (BUN), glucose (GLU), total cholesterol (TC), and triglyceride (TG) in serum were measured by using spectrophotometric kits (Nanjing Jiancheng Bioengineering Institute, Nanjing, China). The concentrations of serum immunoglobulin A (IgA), immunoglobulin G (IgG), and immunoglobulin M (IgM) were analyzed by using ELISA kits (Nanjing Jiancheng Bioengineering Institute, Nanjing, China).

### Rumen Fermentation Index Analysis

At the end of the trial, five lambs from each group were randomly selected and euthanized via electrical stunning. Within 30 min postmortem, rumen fluid was collected and filtered through a four-layer cheesecloth, and the pH value was determined by using a portable pH meter immediately (PHS-3C, Shanghai, China). At the same time, 1 ml of ruminal fluid was preserved at −80° for DNA extraction; other samples were processed to analyze VFA, microbial protein (MCP), and ammonia–N (NH_3_-N). The concentrations of VFA were determined by a gas chromatograph (Agilent Technologies 7820A, USA) based on the method reported previously (14); the ruminal MCP concentration was detected using a spectrophotometric method, and the concentration of NH_3_-N was detected using a uric acid assay kit (Nanjing Jiancheng Bioengineering Institute, Nanjing, China) according to the manufacturer's instructions.

### DNA Extraction, PCR Amplification of 16S rRNA, and Sequencing

DNA extraction, genomic library construction, and sequencing of rumen microorganisms were carried out by Shanghai Meiji Biomedical Technology Co., Ltd. Briefly, total DNA was extracted from rumen samples, and then, the universal primers of bacteria (338F: 5′-ACTCCTRCGGGAGGCAGCAG-3′ and 806R: 5′-GGACTACHVGGGTWTCTAAT-3′) were used to amplify the V3–V4 regions of 16S ribosomal RNA (rRNA). The amplification was initiated with denaturation at 94°C for 3 min, 30 cycles of denaturation at 94°C for 30 s, 58°C for 30 s, 72°C for 90 s, and a final extension at 72°C for 5 min. Finally, The amplified products were electrophoresed on a 2% (w/v) agarose gel and recovered using an AxyPrep DNA Gel Extraction Kit (Axygen, Shanghai, China). The purified effects were quantified by QuantiFlour TM-ST fluorimeter (Promega, Beijing, China). A composite sequencing library was generated by pooling in equimolar ratios of amplicons and sent for paired-end sequencing (2 × 300 bp) on an Illumina Miseq platform at Major Bio-Pharm Technology Co., Ltd. (Shanghai, China).

### Bioinformatics Analysis

Operational taxonomic units (OTUs) were clustered with a 97% similarity cutoff from the clean Fastq data, and chimeric sequences were identified and removed using Usearch 7.0 (http://drive5.com/usearch/). These OTUs were used for diversity (Shannon and Simpson), richness (Ace and Chao), and rarefaction curve analysis using Mothur 1.30.2 (https://www.mothur.org/wiki/Download_mothur). Representative sequences of OTUs were aligned to the SILVA database (https://www.arb-silva.de) for bacteria taxonomic assignments using Qiime (http://qiime.org/install/index.html).

### Statistical Analysis

Basic record statistics were performed in Excel, and all data were statistically analyzed by one-way ANOVA with SPSS 23.0 software. Duncan's test was used to compare differences among the treatment groups. The level of statistical significance was present at *P* < 0.05 and 0.05 ≤ *P* < 0.10 was considered as a tendency.

## Result

### Growth Performance

As shown in Table 2, there was no difference between the initial weight and final weight, but a trend of decline was found in the average daily feed intake (ADFI) of lambs with compound treatment compared with the control (0.05 ≤ *P* < 0.10). Supplementation with CMP significantly increased the average daily gain (ADG) and decreased the ratio of F:G compared with the control group (*P* < 0.05). In addition, the ADG of lambs in probiotics and compound groups was lower than that in the CMP group, while there was no difference from the control group.

**Table 2:** Effects of probiotics and CMP on growth performance in lambs.

**Items**	**Control**	**Probiotics**	**CMP**	**Compound**	**SEM**	***P*-value**
Initial weight (kg)	22.59	21.49	21.12	21.59	0.46	0.736
Final weight (kg)	26.34	25.01	26.68	25.74	0.51	0.685
ADFI (g/day)	775.07	734.82	755.24	711.89	8.59	0.057
ADG (g/day)	62.59^b^	58.59^b^	92.68^a^	69.17^b^	4.06	0.011
F/G	14.13^a^	11.67^ab^	8.85^b^	11.30^ab^	0.66	0.044

*^a, b^Means within a row with no common superscript differ significantly (P < 0.05)*.

*Control: the basal diet (n = 9); probiotics: supplemented with 0.1% probiotics (consists of Bacillus licheniformis, Bacillus subtilis, and Lactobacillus plantarum at a ratio of 1:1:0.5) in the basal diet (n = 10); CMP: supplemented with 0.1% Chinese medicine polysaccharides in the basal diet, which come from the mixture of Lycium barbarum and Astragalus membranaceus in the ratio of 2:1, and in which the content of polysaccharides was 114.7 mg/g (n = 9); compound: supplemented with 0.1% probiotics and 0.1% Chinese medicine polysaccharides in the basal diet (n = 10)*.

*Initial weight and final weight were analyzed with lambs as the experimental unit. ADG, ADMI, and F/G were analyzed with pen as the experimental unit*.

*ADG, average daily gain; ADFI, average dry feed intake; F/G, feed to gain ratio*.

### Serum Biochemical Indices

As shown in Table 3, CMP treatment significantly increased the contents of IgA, IgG, and IgM in serum, but probiotics treatment only increased the concentrations of IgG compared with the control (*P* < 0.05). Moreover, both probiotics and compound treatments significantly increased the concentrations of GLU, while there was no difference in the contents of TP, ALB, GLB, BUN, TG, and TC among all groups.

**Table 3:** Effects of probiotics and CMP on serum biochemical indices in lambs.

**Items**	**Control**	**Probiotics**	**CMP**	**Compound**	**SEM**	***P*-value**
TP (g/L)	72.69	72.98	74.51	71.54	0.83	0.691
ALB (g/L)	27.70	27.42	26.68	28.08	0.54	0.844
GLB (g/L)	44.99	45.56	47.83	43.46	0.61	0.070
BUN (mmol/L)	6.03	6.75	7.68	7.01	0.31	0.314
GLU (mmol/L)	3.86^b^	4.41^a^	3.65^b^	4.33^a^	0.09	0.001
TG (mmol/L)	0.32	0.27	0.30	0.30	0.01	0.463
TC (mmol/L)	2.49	2.32	2.38	2.32	0.09	0.907
IgA/(g/L)	0.48^b^	0.48^b^	0.54^a^	0.45^b^	0.01	0.025
IgG/(g/L)	20.91^b^	24.17^a^	23.22^a^	19.18^b^	0.57	0.001
IgM/(g/L)	1.25^b^	1.25^ab^	1.28^a^	1.24^b^	0.01	0.035

*^a, b^Means within a row with no common superscript differ significantly (P < 0.05)*.

*Control: the basal diet (n = 5); probiotics: supplemented with 0.1% probiotics (consists of Bacillus licheniformis, Bacillus subtilis, and Lactobacillus plantarum at a ratio of 1:1:0.5) in the basal diet; CMP: supplemented with 0.1% Chinese medicine polysaccharides in the basal diet, which come from the mixture of Lycium barbarum and Astragalus membranaceus in the ratio of 2:1, and in which the content of polysaccharides was 114.7 mg/g; compound: supplemented with 0.1% probiotics and 0.1% Chinese medicine polysaccharides in the basal diet*.

*All traits in this table were analyzed with cattle as the experimental unit*.

T*P, total protein; ALB, albumin; GLB, globulin; GLB, TP-ALB; BUN, blood urea nitrogen*.

*GLU, blood glucose; TC, total cholesterol; TG, triglyceride*.

I*gA, immunoglobulin A; IgG, immunoglobulin G; IgM, immunoglobulin M*.

### Rumen Fermentation Parameters

As shown in Table 4, probiotics treatment reduced the pH value in the ruminal fluid of lambs but increased the concentration of MCP compared with the control (*P* < 0.05). The ratio of acetate acid and propionic acid in the CMP group was declined compared with the control (*P* < 0.05). Moreover, the compound (probiotics plus CMP) treatment decreased the value of pH and the ratio of acetate and propionic acid in ruminal fluid compared with the control (*P* < 0.05).

**Table 4:** Effects of probiotics and CMP on rumen fermentation in lambs.

**Items**	**Control**	**Probiotics**	**CMP**	**Compound**	**SEM**	***P*-value**
pH value	6.85^a^	6.63^b^	6.87^a^	6.66^b^	0.03	0.002
NH_3_-N, mg/100 ml	24.59	23.27	21.43	17.25	1.05	0.058
MCP, mg/ml	0.21^b^	0.54^a^	0.20^b^	0.18^b^	0.04	<0.001
acetic acid, mmol/L	29.42	25.87	24.63	31.04	1.55	0.442
propionic acid, mmol/L	8.16	7.61	9.03	9.46	0.41	0.383
butyric acid, mmol/L	6.24	6.90	6.23	5.66	0.34	0.657
Acetic acid/propionic acid	3.63^a^	3.38^ab^	2.74^c^	3.23^b^	0.10	0.001
T-VFA, mmol/L	43.82	40.37	39.90	46.16	2.11	0.710

*^a, b^Means within a row with no common superscript differ significantly (P < 0.05)*.

*Control: the basal diet (n = 5); probiotics: supplemented with 0.1% probiotics (consists of Bacillus licheniformis, Bacillus subtilis, and Lactobacillus plantarum at a ratio of 1:1:0.5) in the basal diet; CMP: supplemented with 0.1% Chinese medicine polysaccharides in the basal diet, which come from the mixture of Lycium barbarum and Astragalus membranaceus in the ratio of 2:1, and in which the content of polysaccharides was 114.7 mg/g; compound: supplemented with 0.1% probiotics and 0.1% Chinese medicine polysaccharides in the basal diet*.

*All traits in this table were analyzed with cattle as the experimental unit (n = 5)*.

*NH3–N, ammonia–N; MCP, microbial protein; T-VFA, total volatile fatty acid*.

### Diversity of Ruminal Bacteria

**Figure 1:**
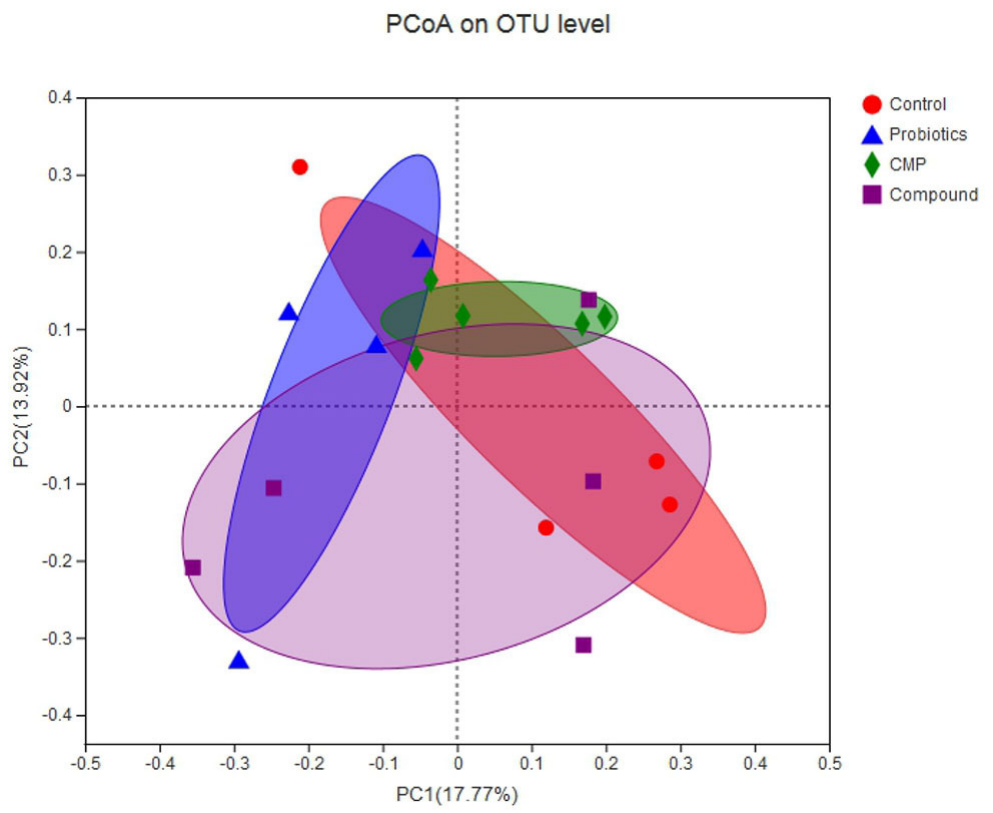
Principal coordinates analysis (PCoA) diagram of rumen microbial community structure across groups. Control: the basal diet (*n* = 4); probiotics: supplemented with 0.1% probiotics (consists of *Bacillus licheniformis, Bacillus subtilis*, and *Lactobacillus plantarum* at a ratio of 1:1:0.5) in the basal diet (*n* = 4); CMP: supplemented with 0.1% Chinese medicine polysaccharides in the basal diet, which come from the mixture of *Lycium barbarum* and *Astragalus membranaceus* in the ratio of 2:1, and in which the content of polysaccharides was 114.7 mg/g (*n* = 5); compound: supplemented with 0.1% probiotics and 0.1% Chinese medicine polysaccharides in the basal diet (*n* = 5).

In this experiment, a total of 931,700 valid sequences were generated from 18 samples, and the richness and alpha diversity of the community were analyzed by Ace, Chao, Shannon, and Simpson indices in Table 5. However, there were no differences in these indicators (*P* > 0.05). As shown in Figure 1, Principal coordinates analysis (PCoA) axes 1 and 2 accounted for 17.77% and 13.92% of the total variation, respectively. However, there were no significant differences in the ruminal bacterial community structure between the treatments and control group.

**Table 5:** Effects of probiotics and CMP on bacterial richness and diversity indices in the rumen of captive lambs.

**Items**	**Control**	**Probiotics**	**CMP**	**Compound**	**SEM**	***P*-value**
OTU	720.00	590.25	720.20	676.80	24.96	0.254
Shannon index	4.38	4.32	4.76	4.41	0.07	0.132
Simpson index	0.05	0.05	0.02	0.05	0.01	0.332
Ace index	889.25	734.84	876.34	832.17	28.11	0.244
Chao index	888.21	732.47	880.92	833.63	28.70	0.237
coverage	0.9924	0.9938	0.9928	0.9931	0.00	0.278

*Control: the basal diet (n = 4); probiotics: supplemented with 0.1% probiotics (consists of Bacillus licheniformis, Bacillus subtilis, and Lactobacillus plantarum at a ratio of 1:1:0.5) in the basal diet (n = 4); CMP: supplemented with 0.1% Chinese medicine polysaccharide in the basal diet, which come from the mixture of Lycium barbarum and Astragalus membranaceus in the ratio of 2:1, and in which the content of polysaccharides was 114.7 mg/g (n = 5); compound: supplemented with 0.1% probiotics and 0.1% Chinese medicine polysaccharides in the basal diet (n = 5)*.

*OTU, operational taxonomic units, which were screened for further annotation, and sequences with > 97% similarity were assigned to the same OTUs*.

**Figure 2:**
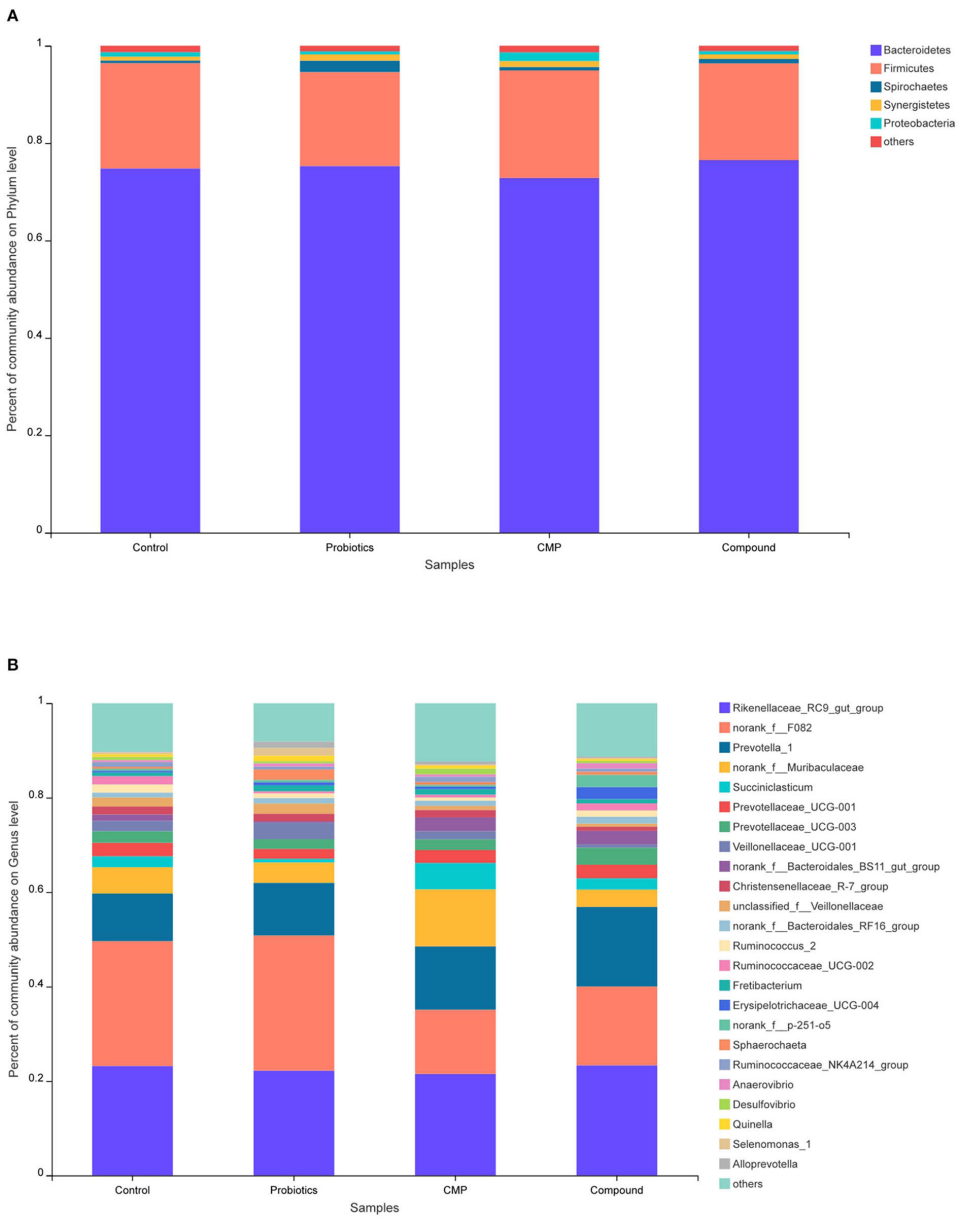
Relative abundance distribution of rumen flora at **(A)** phylum and **(B)** genus levels. Control: the basal diet (*n* = 4); probiotics: supplemented with 0.1% probiotics (consists of *Bacillus licheniformis, Bacillus subtilis*, and *Lactobacillus plantarum* at a ratio of 1:1:0.5) in the basal diet (*n* = 4); CMP: supplemented with 0.1% Chinese medicine polysaccharides in the basal diet, which come from the mixture of *Lycium barbarum* and *Astragalus membranaceus* in the ratio of 2:1, and in which the content of polysaccharides was 114.7 mg/g (*n* = 5); compound: supplemented with 0.1% probiotics and 0.1% Chinese medicine polysaccharides in the basal diet (*n* = 5).

The relative abundance of rumen microbiota is displayed at the phylum level (Figure 2A and Table 6) and the genus level (Figure 2B and Table 7). Results in Table 6 show that supplementation with probiotics tended to reduce the relative abundance of bacteria belonging to phylum *Kiritimatiellaeota* (0.05 ≤ *P* < 0.10) compared with the control, and reduced the relative abundance of phylum *Fibrobacteres* compared with the compound group but increased the relative abundance of phylum *Bacteria_NA* compared with CMP and compound groups (*P* < 0.05). There was no difference found on other phyla among groups. At the genus level in Table 7, the relative abundance of *unclassified_f__Veillonellaceae* with probiotics treatment was increased, the relative abundance of *norank_f__Muribaculaceae* and *Succiniclasticum* in the CMP group were enhanced, and the relative abundance of *Ruminococcaceae_UCG-002* with compound treatment was increased compared with the control group (*P* < 0.05). In addition, the relative abundance of *Veillonellaceae_UCG-001* in the probiotics group was raised compared with the CMP and compound groups (*P* < 0.05).

**Table 6:** Effects of probiotics and CMP on rumen bacterial flora structure (phylum level) %.

**Items**	**Control**	**Probiotics**	**CMP**	**Compound**	**SEM**	***P*-value**
Bacteroidetes	74.78	75.28	72.86	76.52	1.13	0.720
Firmicutes	21.66	19.30	22.06	19.87	1.11	0.812
Proteobacteria	0.90	1.02	1.61	0.69	0.17	0.286
Synergistetes	0.79	1.27	1.20	0.92	0.17	0.765
Spirochaetes	0.54	0.78	0.72	0.90	0.12	0.810
Patescibacteria	0.30	0.23	0.26	0.25	0.03	0.931
Tenericutes	0.22	0.13	0.20	0.32	0.04	0.353
Actinobacteria	0.17	0.32	0.25	0.12	0.06	0.652
Kiritimatiellaeota	0.29	0.13	0.18	0.18	0.02	0.058
Bacteria_NA	0.16^ab^	0.24^a^	0.09^b^	0.04^b^	0.03	0.034
Chloroflexi	0.09	0.04	0.04	0.05	0.01	0.122
Fibrobacteres	0.06^ab^	0.03^b^	0.03^b^	0.09^a^	0.01	0.038
Cyanobacteria	0.02	0.02	0.02	0.03	0.00	0.976
Epsilonbacteraeota	0.01	0.00	0.01	0.01	0.00	0.647
Lentisphaerae	0.01	0.01	0.00	0.01	0.00	0.740
Elusimicrobia	0.01	0.01	0.00	0.01	0.00	0.870

^a, b^*Means within a row with no common superscript differ significantly (P < 0.05)*.

*Control: the basal diet (n = 4); probiotics: supplemented with 0.1% probiotics (consists of Bacillus licheniformis, Bacillus subtilis, and Lactobacillus plantarum at a ratio of 1:1:0.5) in the basal diet (n = 4); CMP: supplemented with 0.1% Chinese medicine polysaccharide in the basal diet, which come from the mixture of Lycium barbarum and Astragalus membranaceus in the ratio of 2:1, and in which the content of polysaccharides was 114.7 mg/g (n = 5); compound: supplemented with 0.1% probiotics and 0.1% Chinese medicine polysaccharides in the basal diet (n = 5)*.

**Table 7:** Effects of probiotics and CMP on rumen bacterial flora structure (genus level) %.

**Items**	**Control**	**Probiotics**	**CMP**	**Compound**	**SEM**	***P*-value**
Rikenellaceae_RC9_gut_group	23.19	22.21	21.50	23.33	1.91	0.987
norank_f__F082	26.43	28.61	13.59	16.69	2.46	0.071
Prevotella_1	10.06	11.14	13.44	16.85	1.73	0.552
norank_f__Muribaculaceae	5.59^b^	4.31^b^	10.76^a^	3.67^b^	0.95	0.009
Succiniclasticum	2.32^b^	0.78^b^	5.58^a^	2.39^b^	0.54	0.003
Prevotellaceae_UCG-001	2.85	2.10	2.74	2.87	0.33	0.862
Prevotellaceae_UCG-003	2.46	2.06	2.27	3.60	0.35	0.394
Veillonellaceae_UCG-001	2.18^ab^	3.49^a^	1.75^b^	0.70^b^	0.33	0.011
norank_f__Bacteroidales_BS11_gut_group	0.43	0.30	0.67	0.74	0.14	0.688
Christensenellaceae_R-7_group	1.68	1.60	1.57	1.05	0.15	0.417
unclassified_f__Veillonellaceae	0.73^b^	2.17^a^	0.88^b^	0.63^b^	0.23	0.043
norank_f__Bacteroidales_RF16_group	1.01	1.15	1.08	1.47	0.21	0.886
Ruminococcaceae_UCG-002	0.60^b^	0.43^b^	0.59^b^	1.75^a^	0.18	0.013
Fretibacterium	0.77	1.27	1.19	0.91	0.17	0.755
Erysipelotrichaceae_UCG-004	0.35	0.64	0.52	0.69	0.10	0.710
Sphaerochaeta	0.39	0.63	0.59	0.68	0.12	0.879
Ruminococcaceae_NK4A214_group	0.97	0.74	1.13	0.74	0.08	0.268
Ruminococcaceae_UCG-010	0.83	0.87	0.66	0.60	0.10	0.799
Ruminococcus_2	0.44	0.61	0.67	0.48	0.07	0.693
norank_f__p-251-o5	0.44	0.53	0.38	0.62	0.08	0.786

*^a, b^Means within a row with no common superscript differ significantly (P < 0.05)*.

*Control: the basal diet (n = 4); probiotics: supplemented with 0.1% probiotics (consists of Bacillus licheniformis, Bacillus subtilis, and Lactobacillus plantarum at a ratio of 1:1:0.5) in the basal diet (n = 4); CMP: supplemented with 0.1% Chinese medicine polysaccharide in the basal diet, which come from the mixture of Lycium barbarum and Astragalus membranaceus in the ratio of 2:1, and in which the content of polysaccharides was 114.7 mg/g (n = 5); compound: supplemented with 0.1% probiotics and 0.1% Chinese medicine polysaccharides in the basal diet (n = 5)*.

## Discussion

In this study, dietary supplementation of CMP increased the average daily gain of lambs but decreased the ratio of feed to gain, which indicated that CMP is composed of *Astragalus* polysaccharides (APS) and *L. barbarum* polysaccharides (LBPs) that could improve the growth performance of lambs. Dietary supplementation with Chinese herbal polysaccharides, such as *A. membranaceus* polysaccharide (AMP), *Ginseng* polysaccharide, and *L. barbarum* polysaccharide, has been used extensively to improve growth performance in broiler chickens and piglets (15, 16). There are few reports noticed about the effect of Chinese medicine polysaccharides on performance in ruminant. Nevertheless, a previous study showed that a diet with *Astragalus* polysaccharides and *A. membranaceus* root could improve antioxidant capacity and affect rumen fermentation patterns of lambs (17). A similar result indicated that APS increased the total VFA production of lambs (18). Another recent report demonstrated that a diet with bee pollen polysaccharide improved nutrient digestibility of calf (19). Hence, the enhancement of growth performance in this study may be owed to the improving nutrient utilization and antioxidant capacity of lambs fed diets supplemented with CMP.

Similarly, the addition of CMP significantly increased the contents of immunoglobulin in serum, which indicated that CMP treatment could improve the immune function of lambs. Immunoglobulin is a kind of non-specific immune molecule in animals. There are three main types of immunoglobulin in blood, including IgA, IgM, and IgG. The polysaccharides from natural plants can possess activity in promoting lymphocyte proliferation and improving the expression of cytokines to enhance immunity (20). A previous study revealed that administration of APS increased the level of IgG and IgM in serum (21). It has been reported that APS might induce the differentiation of splenic DCs with the enhancement of T lymphocyte immune function *in vitro* (22). Furthermore, in agreement with the current experiment, LBP supplementation in the broilers diet promoted humoral immune response leading to the increase in serum IgA and IgG concentrations (12), which may be due to LBP-activated macrophages to generate nitric oxide and promote cytokine secretion (23).

Although supplemental probiotics did not improve the average daily feed intake and gain of lambs, serum IgG and GLU concentrations were enhanced in the probiotics group. Similar to our results, Jia (24) also observed supplemental probiotics consisting of *Bacillus lichens* and *Saccharomyces cerevisiae* increased IgG content in lambs. The enhancement of serum GLU concentration could be due to the improvement of probiotics that produce nutrients and growth factors that are stimulatory to beneficial microorganisms of the gut microbiota. However, in contrast to our results, Ibrahim reported that GLU was not affected by probiotics supplementation (25). Another report by Ahmed pointed that GLU was significantly lowered on the 30th day in the probiotic (*B. subtilis*) supplementation group of growing Barki lambs (26). This may be due to differences in the activities of the probiotic strains and survivability throughout the gut, which appear to be of great importance for optimal efficacy (13).

Low ruminal pH can be caused by an accumulation of ruminal VFA (27). The present results showed that a lower ruminal pH was observed in the probiotics and compound groups, which was possibly due to the addition of *L. plantarum* to feed, promoting the ferment to produce more lactic acid. Consistent with our results, a previous study revealed that the pH value in the rumen was decreased after feeding *Bacillus natto* to the cows (28). Besides, supplemental probiotics increased significantly the content of MCP, which indicated that probiotics promoted rumen microorganisms to synthesize more MCP using ammonia nitrogen. Consistently, preweaning calves fed *L. plantarum 299v* exhibited an increase in MCP concentrations than those in the control group (29).

VFAs mainly include acetic acid, propionic acid, and butyric acid. The concentrations of VFA contribute to ruminants' health by sustaining the rumen ecosystem and acting as an energy source, and the ratio of acetic acid and propionic acid can reflect the rumen fermentation mode. The present result about reducing the ratio of acetate and propionic acid with CMP and compound treatment indicated that diet with Chinese medicine polysaccharide improved rumen fermentation mode. In line with the result, adding 15 g/kg *Astragalus* polysaccharides to the lamb diet increased the concentration of propionic acid and reduced the ratio of acetate and propionic acid in rumen fluid (11). Ammonia–nitrogen (NH_3_-N) in the rumen is the main nitrogen source for the synthesis of microbial protein, and its normal concentration range is 6.3–27.5 mg/dl (30). In this experiment, the concentrations of NH_3_-N in all groups were within the range, but the concentrations of rumen NH_3_-N had a declining trend in the compound group, which suggested that diet with the compound of probiotics and Chinese medicine polysaccharide might reduce the activity of rumen proteolytic enzyme. A similar report showed that probiotics supplementation decreased the concentration of NH_3_-N of growing lambs significantly (31). Another report by Wang indicated that a diet with traditional Chinese medicine compound 1 (TCMC 1) tends to decrease the concentration of NH_3_-N (32).

In this study, there were 931,700 valid sequences in the rumen of lambs, and the coverage rate was over 99%, which indicated that the sequencing results could basically reflect most species in the rumen bacterial flora of lambs. Based on the Silva taxonomic database and using the analysis program Qiime, 1,505 bacterial OTUs were classified and assigned to 16 phyla and 197 genera in the present study. The present results showed the microbial community of lambs was dominated by *Bacteroidetes* and *Firmicutes* on the phylum level regardless of group. The results are consistent with the study by (33, 34), in which *Bacteroidetes, Firmicutes*, and *Proteobacteria* were found predominantly in rumen fluid. However, the phylum compositions of *Fibrobacteres* in the compound group were higher than that in probiotics and CMP groups. *Fibrobacteres* played an essential role in fiber degradation and utilized cellulose to provide nutrients for ruminants, and the current results may be due to the interaction of probiotics and CMP. Previous reports suggested that Chinese herb extracts fermented with probiotic bacteria may increase the oxidative stability and antibacterial activity, which may be due to probiotics promoting the absorption of Chinese herbs in animals, and at the same time, Chinese herbs provided nutrients for probiotics and promoted their proliferation (35, 36).

At the genus level, *Rikenellaceae_RC9_gut_group, norank_f__F082*, and *Prevotella_1* were the dominant bacteria in the four diet groups. No difference was observed in these bacteria, but CMP treatment increased the content of *Succiniclasticum* and *norank_f__Muribaculaceae* in rumen fluid. *Succiniclasticum* isolated by Van Gylswyk (37) from the bovine rumen, which belongs to *Firmicutes*, can produce succinic acid and convert it into propionic acid and further make glucose. The present results suggest that a diet with CMP might increase the degradation of fiber in the rumen and change the rumen fermentation mode, which was consistent with the results of the decrease in acetic acid/propionic acid and the increase in daily gain of lambs. *Ruminococcaceae* is one of the *Firmicutes* that can produce cellulase and hemicellulase to degrade plant fibers and is the main cellulolytic bacterium. This study showed that both probiotics and CMP treatments did not affect the relative abundance of *Ruminococcaceae_UCG-002*, but the diet with the compound of probiotics and CMP increased the relative abundance of the species, which suggested that there may be an interaction between polysaccharides and probiotics on promoting *Ruminococcaceae_UCG-002* reproduction in the rumen, and increasing the degradation of cellulose in the rumen improve the utilization rate of feed. Although all treatments did not change the relative abundance of the *Veillonellaceae* significantly compared with the control, this abundance in the probiotics group was higher than those in the CMP and compound groups. *Veillonellaceae* belongs to *Firmicutes*, which can degrade and utilize cellulose. However, different from our experimental results, Chae reported that the relative abundance of *Veillonellaceae* was reduced after adding probiotic *Enterococcus faecium NCIMB 11181* to the diet of the weaned pig (38). Schofield found no significant difference in the rumen community in sheep as a result of feeding the probiotic *Bacillus amyloliquefaciens H57* (3). This difference may be due to the variety and dosage of probiotics. The rumen bacterial community is extremely complex, resulting from the interaction of external factors and animals themselves, and its influencing mechanism needs to be further studied.

## Conclusion

Diet with probiotics can promote protein fermentation, but the diversity of bacteria had a decreasing trend. Supplementation with CMP increased the relative abundance of Fibrobacteria, changed the rumen fermentation mode, and improved the immune function and growth performance.

## Data Availability Statement

The data presented in the study are deposited in the Sequence Read Archive (SRA) of National Center for Biotechnology Information (NCBI) repository, and accession number (SRA accession number) is PRJNA707607.

## Ethics Statement

The animal study was reviewed and approved by Committee for the Care and Use of Experimental Animals at Jiangxi Agricultural University.

## Author Contributions

XS, HC, and BG designed the overall study. BG, HC, MY, JL, YH, and MQ performed the animal feeding experiment and sample analysis. XS and HC wrote the manuscript. All authors contributed to the article and approved the submitted version.

## Conflict of Interest

The authors declare that the research was conducted in the absence of any commercial or financial relationships that could be construed as a potential conflict of interest.
